# Molecular subtypes of breast cancer in Jamaican women: high prevalence of triple-negative and HER2 + disease

**DOI:** 10.1186/s13058-025-02175-7

**Published:** 2026-01-06

**Authors:** Richard Shaw, Derria Cornwall, Simone Badal, Leo-Paul Powell, Ayesha Johnson, Rory Thompson, Patrick Roberts

**Affiliations:** 1 Department of Surgery, Radiology, Anaesthesia, and Intensive Care, Faculty of Medical Sciences, The University of the West Indies, Mona, West Indies Jamaica; 2 Department of Basic Medical Sciences, Faculty of Medical Sciences Teaching and Research Complex, The University of the West Indies, Mona, West Indies Jamaica; 3 College of Nursing, University of South Florida, Florida, USA; 4 Department of Pathology, The University of the West Indies, Mona, Jamaica

**Keywords:** Breast cancer, Jamaican women, Black women, HR + /HER2-, TNBC, Disparity

## Abstract

**Background:**

Breast cancer incidence and mortality patterns vary significantly across populations, with women of African ancestry experiencing disproportionately higher mortality despite lower incidence rates. Jamaica reports concerning breast cancer mortality, yet comprehensive molecular characterization of breast cancer in this predominantly Black Caribbean population remains limited.

**Methods:**

We conducted a retrospective cross-sectional study of 1312 female breast cancer patients diagnosed at the University Hospital of the West Indies in Jamaica between January 2012 and December 2016. Medical records were reviewed for patient demographics, tumour characteristics, and receptor status (estrogen receptor, progesterone receptor, and HER2). Tumours were classified into four molecular subtypes: HR + /HER2- (estrogen receptor positive and/or progesterone receptor positive, HER2 negative), HR + /HER2 + (estrogen receptor positive and/or progesterone receptor positive, HER2 positive), HR-/HER2 + (estrogen receptor negative and progesterone receptor negative, HER2 positive), and triple-negative (HR-/HER2-: estrogen receptor negative and/ progesterone receptor negative, HER2 negative). Bivariate analyses and logistic regression models assessed associations between clinicopathological features and molecular subtypes.

**Results:**

Mean age at diagnosis was 55.1 ± 13.6 years. The predominant tumour characteristics were invasive ductal histology (82%), SBR grade 2 (37%), and tumours ≤ 2 cm (31%). Molecular subtype distribution revealed HR + /HER2- (49%), HR + /HER2 + (13%), HR-/HER2 + (12%), and triple-negative (27%). Notably, total HER2 + disease accounted for 25% of cases. Triple-negative tumours were significantly associated higher grade (AOR = 13.92, *p*  < 0.001), and smaller tumours had reduced odds of being triple negative (AOR = 0.47, *p* < 0.05). Lobular carcinomas showed lower odds of being triple-negative (AOR = 0.17, *p* < 0.01) compared to ductal carcinomas.

**Conclusions:**

Jamaican breast cancer patients exhibit a distinctive molecular profile with a high prevalence of both triple-negative (27%) and HER2 + (25%) disease, which likely contributes to poor outcomes in this population. These findings underscore the need for population-specific approaches to breast cancer management in Jamaica, including universal receptor testing and improved access to targeted therapies, particularly anti-HER2 agents.

**Supplementary Information:**

The online version contains supplementary material available at 10.1186/s13058-025-02175-7.

## Introduction

Breast cancer incidence rates have been consistently increasing over the past decades globally, with wide variations in mortality rates across regions [1]. A notable disparity exists between developed and developing countries: women in less developed regions typically have lower incidence but higher mortality rates, while those in more developed countries experience higher incidence but lower mortality rates [2]. These disparities largely stem from inadequate access to comprehensive treatment options, lack of organized screening programs, and research bias toward populations predominantly residing in developed countries [3].

In the Caribbean region, breast cancer is the leading cause of cancer deaths among women, with Jamaica showing particularly concerning patterns [4]. According to GLOBOCAN 2020 data, the age-standardized incidence rate of breast cancer in Jamaica reached 66.9 per 100,000 women, making it the most commonly diagnosed cancer among Jamaican women [2]. Of greater concern is the mortality rate, which was reported at 28.3 per 100,000 in 2014 [5]. This high mortality-to-incidence ratio contrasts sharply with declining mortality rates observed in developed countries such as the United States and United Kingdom [6–8], suggesting critical gaps in early detection and effective treatment in Jamaica.

The molecular classification of breast cancer has revolutionized our understanding of the disease's heterogeneity and guided targeted therapy approaches. In 2000, Perou et al. [9] introduced landmark intrinsic molecular subtypes based on gene expression profiling: Luminal A (ER-positive, low proliferation), Luminal B (ER-positive, high proliferation), HER2-enriched (HER2-positive, ER-negative), Basal-like (typically triple-negative), and Normal-like subtypes. These classifications demonstrate distinct clinical behaviors, treatment responses, and survival outcomes [10]. Current clinical practice relies on immunohistochemical (IHC) markers—estrogen receptor (ER), progesterone receptor (PR), and human epidermal growth factor receptor 2 (HER2)—to approximate these molecular subtypes, providing clinically relevant classifications that guide therapeutic decisions [11, 12].

Hormone receptor (HR) positive HER2 negative tumours generally have the most favorable prognosis, while triple-negative breast cancers (TNBC)—negative for ER, PR, and HER2—demonstrate the poorest outcomes [13]. TNBC, accounting for approximately 10–15% of breast cancers in most populations, is characterized by aggressive histology, limited treatment options, and significantly shorter survival [14, 15]. Studies have consistently reported higher rates of TNBC among women of African ancestry, with prevalence ranging from 20% to over 50% in various African, Caribbean, and African-American populations [16–18]. Huo et al. demonstrated that while TNBC comprises approximately 16% of breast cancers in White American women, it accounts for 30–40% of cases in African American women [19]. These epidemiological differences suggest complex interactions between genetic ancestry, environmental factors, and social determinants of health [20].

In the Caribbean context, limited data exist regarding the molecular profile of breast cancers. Deloumeaux et al. [21] reported a TNBC prevalence of 14.1% in Guadeloupe, while a more recent study from Barbados found a TNBC rate of 25% [22]. These variations underscore the importance of population-specific studies, as molecular subtype distribution may differ substantially even among predominantly Black populations. Despite having one of the Caribbean's highest breast cancer mortality rates, Jamaica lacks comprehensive data characterizing the molecular subtypes of breast cancer in its population. Such information is critical for understanding the biological factors driving the observed high mortality rates and for developing targeted screening and treatment strategies. Previous studies in Jamaica have been limited by small sample sizes or insufficient molecular characterization [23, 24].

This study aims to assess the prevalence and clinicopathological associations of breast cancer molecular subtypes in a large cohort of Jamaican women. By characterizing the distribution of HR and HER2 status and their associations with clinical and pathological features, we seek to provide essential data to guide population-specific approaches to breast cancer management in Jamaica. Such insights are particularly relevant given established differences in molecular subtype distribution across racial groups and potential implications for tailored screening, prevention, and treatment strategies in the Jamaican healthcare system.

## Methods

### Study design and setting

We conducted a retrospective cross-sectional study of breast cancer patients treated at the University Hospital of the West Indies (UHWI), a major tertiary referral center in Kingston, Jamaica. The UHWI serves patients from across the country, providing specialized oncology care to approximately 40–45% of breast cancer cases nationwide [25]. Data were collected from patient records spanning the period from January 1, 2012, to December 31, 2016, with analysis completed in January 2024. This study was conducted in accordance with the Strengthening the Reporting of Observational Studies in Epidemiology (STROBE) guidelines [26].

### Ethics approval

The study received approval from the Faculty of Medical Sciences Ethics Committee at the University of the West Indies, Mona, Jamaica (ECP38, 2020/2021). Due to the retrospective nature of the study, the requirement for individual patient consent was waived. All data were anonymized during extraction and analysis to ensure patient confidentiality.

### Study population

During the study period, a total of 1365 histologically confirmed breast cancer cases were identified at the UHWI. Of these, 1316 patients (96.4%) had undergone receptor status testing. We included all female patients aged ≥ 18 years with histologically confirmed invasive breast carcinoma who had receptor status testing available. Four male breast cancer cases were excluded, resulting in a final study population of 1312 female patients. Eligibility criteria required availability of basic demographic information, pathological diagnosis, and receptor status (estrogen receptor, progesterone receptor, and HER2) determined by immunohistochemistry. Patients with incomplete receptor status data and recurrent breast cancer cases were also excluded from the analysis.

### Data collection and variables

Data were extracted from the hospital's electronic and paper-based medical records by trained research assistants using a standardized data collection form. Patient demographics included age at diagnosis. Clinical and pathological data encompassed specimen type (core needle biopsy or mastectomy), tumour size, lymph node status, histological type, Scarff-Bloom-Richardson (SBR) grade, and receptor status. Core needle biopsies accounted for 59% (n = 769) of all samples, with the remaining 41% (n = 540) obtained from mastectomy specimens. During the study period, mastectomy was the preferred surgical approach due to limited access to radiation therapy, which is typically required after breast-conserving surgery. Tumour size was categorized according to the TNM classification (8th edition) of the American Joint Committee on Cancer [27]: T1 (tumours ≤ 2 cm), T2 (tumours 2–5 cm), and T3 (tumours > 5 cm). T4 tumours (any size with chest wall or skin involvement) were noted but grouped with T3 for analytical purposes due to limited numbers. For multifocal or multicentric tumours, the size of the largest tumour was recorded. Histological classification followed the World Health Organization criteria [28], categorizing tumours as invasive ductal carcinoma, invasive lobular carcinoma, or other histological types (including mixed ductal and lobular, mucinous, medullary, and other rare variants). SBR grading was based on the modified Scarff-Bloom-Richardson system [29], categorizing tumours as grade 1 (well-differentiated), grade 2 (moderately differentiated), or grade 3 (poorly differentiated). Clinical and pathological data encompassed specimen type (core needle biopsy or mastectomy), tumour size, lymph node status, histological type, Scarff-Bloom-Richardson (SBR) grade, and receptor status including hormone receptor (HR) status (estrogen receptor and progesterone receptor) and human epidermal growth factor receptor 2 (HER2) status.

### Immunohistochemical analysis

Estrogen receptor (ER), progesterone receptor (PR), and HER2 status were determined by immunohistochemistry (IHC) on formalin-fixed, paraffin-embedded tissue sections at the UHWI Department of Pathology according to standard protovols [11, 30]. ER and PR status were assessed using antibodies against ER (clone SP1) and PR (clone PgR636). Nuclear staining was scored using the Allred scoring system [8], combining percentage of positive cells (proportion score 0–5) and staining intensity (intensity score 0–3) for a total score of 0–8. Tumours with a total score ≥ 3 were classified as receptor-positive, while those with scores < 3 were considered negative. HER2 status was evaluated using the HercepTest™ (Dako). Membranous staining was scored 0 to 3 + : 0 (no staining or incomplete staining in ≤ 10% of tumour cells), 1 + (faint incomplete membrane staining in > 10% of tumour cells), 2 + (weak to moderate complete membrane staining in > 10% of tumour cells), and 3 + (strong complete membrane staining in > 10% of tumour cells). Cases with equivocal results (2 +) underwent fluorescence in situ hybridization (FISH) testing. A positive FISH result was defined as HER2/CEP17 ratio ≥ 2.0 or average HER2 copy number ≥ 6.0 signals per cell. Tumours were categorized as HER2-positive if they scored 3 + by IHC or demonstrated HER2 gene amplification by FISH.

### Molecular subtype classification

Based on the receptor status results, tumours were classified into four molecular subtypes that approximate the intrinsic subtypes defined by gene expression profiling [9, 31]: i) Hormone receptor positive/HER2 negative (HR + /HER2-): ER + and/or PR + , HER2-; ii) Hormone receptor positive/HER2 positive (HR + /HER2 +): ER + and/or PR + , HER2 + ; iii) Hormone receptor negative/HER2 positive (HR-/HER2 +): ER- and PR-, HER2 + and iv) Triple-negative (HR-/HER2-): ER- and PR-, HER2-. This classification aligns with current clinical practice and treatment decision-making guidelines [32, 33].

### Statistical analysis

Statistical analyses were performed using SPSS version 26.0 (IBM Corp., Armonk, NY, USA) and Stata version 16.0 (StataCorp, College Station, TX, USA). Categorical variables were presented as frequencies and percentages. Continuous variables were reported as means with standard deviations (SD) for normally distributed data or medians with interquartile ranges (IQR) for non-normally distributed data. For bivariate analyses, we used Pearson's chi-square test or Fisher's exact test (when expected cell counts were < 5) to examine associations between categorical variables. The Kruskal–Wallis test was used to compare age distributions across tumour characteristic categories. To quantify the associations between clinicopathological features and outcomes of interest (molecular subtypes and lymph node status), we estimated a multinomial logistic regression model for molecular subtype, and a binary logistic regression model for lymph node status. Models included all variables with significant bivariate associations. Results were presented as adjusted odds ratios (AOR) with 95% confidence intervals (CI). Statistical significance was set at p < 0.05. To address potential bias due to missing data, we conducted sensitivity analyses comparing the characteristics of patients with complete versus incomplete data. Missing data patterns were evaluated, and multiple imputation techniques were considered when appropriate for variables with > 10% missing values. However, for the primary analyses, we employed a complete-case approach, excluding cases with missing values for the variables under examination.

Statistical analyses were performed using SPSS version 26.0 (IBM Corp., Armonk, NY, USA) and Stata version 16.0 (StataCorp, College Station, TX, USA). Categorical variables were presented as frequencies and percentages. Continuous variables were reported as means with standard deviations (SD) for normally distributed data or medians with interquartile ranges (IQR) for non-normally distributed data. For bivariate analyses, we used Pearson's chi-square test or Fisher's exact test (when expected cell counts were < 5) to examine associations between categorical variables. The Kruskal–Wallis test was used to compare age distributions across tumour characteristic categories. To quantify associations between clinicopathological features and outcomes of interest (molecular subtypes and lymph node status), we estimated multinomial logistic regression for molecular subtype and binary logistic regression for lymph node status. Models included all variables with significant bivariate associations. Results were presented as adjusted odds ratios (AOR) with 95% confidence intervals (CI). Statistical significance was set at p < 0.05. To address potential bias due to missing data, we conducted sensitivity analyses comparing characteristics of patients with complete versus incomplete data. Missing data patterns were evaluated, and multiple imputation techniques were considered when appropriate for variables with > 10% missing values. However, for primary analyses, we employed a complete-case approach, excluding cases with missing values for the variables under examination.

## Results

### Patient and tumour characteristics

Of 1365 women diagnosed with invasive breast carcinoma at the University Hospital of the West Indies between January 1, 2012, and December 31, 2016, 53 (3.9%) were excluded due to unavailable receptor status, resulting in a final cohort of 1312 patients for analysis. Table 1 summarizes the demographic and pathological characteristics of the study population. The mean age at diagnosis was 55.1 ± 13.6 years (range 21–93 years), with the majority of patients (52%) diagnosed between ages 40–59 years. Only 12% of patients were diagnosed before age 40, while 36% were diagnosed at age 60 or older (Table 1). Core needle biopsies accounted for 59% (n = 769) of all samples, with the remaining 41% (n = 540) obtained from mastectomy specimens. Regarding tumour size, 31% of tumours were classified as T1 (≤ 2 cm), 24% as T2 (2–5 cm), and 12% as T3 (> 5 cm), with 33% having no available tumour size data (Table 1). Positive lymph node status was recorded in 29% of cases. The predominant histological type was invasive ductal carcinoma (82%), with invasive lobular carcinoma accounting for only 8%. SBR grading was available for 70% of tumours, with grade 2 being most common (37%), followed by grade 3 (21%) and grade 1 (12%). For receptor status, 52.8% of tumours were estrogen receptor positive (ER +), 47.7% were progesterone receptor positive (PR +), and 24.0% were human epidermal growth factor receptor 2 positive (HER2 +). Based on combined hormone receptor (HR) and HER2 status, the molecular subtypes were distributed as follows: HR + /HER2- (49%), HR + /HER2 + (13%), and HR-/HER2 + (12%), and triple-negative (HR-/HER2-) (27%) (Table 1).

**Table 1:** Demographic and Pathological Characteristics of Jamaican Breast Cancer Patients (N = 1312)

Characteristic	Frequency (n)	Percentage (%)
Age (years)		
Under 40	157	12
40–49	340	26
50–59	339	26
60–69	258	20
70–79	144	11
80 & over	61	5
Mean ± SD	55.1 ± 13.6	
Range	21–93	
Specimen Type		
Core needle biopsy	769	59
Mastectomy	540	41
Tumour Size (cm)		
T1 (≤ 2 cm)	401	31
T2 (2–5 cm)	318	24
T3 (> 5 cm)	155	12
Not available	437	33
Lymph Node Status		
Negative	934	71
Positive	378	29
Histological Type		
Invasive ductal	1072	82
Invasive lobular	103	8
Other	137	10
SBR Grade		
1	163	12
2	482	37
3	273	21
Not available	392	30
Receptor Status		
ER +	693	52.8
PR +	626	47.7
HER2 +	319	24.0
Molecular Subtype		
HR + /HER2-	638	49
HR + /HER2 +	165	13
HR-/HER2 +	154	12
HR-/HER2- (Triple-negative)	353	27

SBR: Scarff-Bloom-Richardson; ER: Estrogen receptor; PR: Progesterone receptor; HER2: Human epidermal growth factor receptor 2; HR: Hormone receptor (ER and/or PR)

This comprehensive table presents the baseline demographic and clinical characteristics of all 1312 patients included in the study. It displays the distribution of age at diagnosis (with both categorical and continuous measures), specimen types, tumour size according to TNM classification, lymph node status, histological tumour types, Scarff-Bloom-Richardson (SBR) grading, hormone receptor status (ER and PR), HER2 status, and molecular subtypes. The table highlights that most patients were diagnosed between ages 40–59 years, with invasive ductal carcinoma as the predominant histological type, and HR + /HER2- as the most common molecular subtype (49%), followed by triple-negative breast cancer (27%)

### Association with age

Single-predictor models revealed minimal associations between age at diagnosis and tumour characteristics (data not shown). Patients with SBR grade 3 tumours were slightly younger (mean age 53 years) than those with grade 1 or 2 tumours (mean age 55 years) (p = 0.038). No statistically significant age differences were observed across molecular subtypes or lymph node status.

### Associations with molecular subtypes

Bivariate tests of association identified significant associations between molecular subtypes and some clinicopathological characteristics. Molecular subtypes significantly varied according to specimen type. Multinomial Logistic regression confirmed that mastectomy specimens had equal odds of being HR + /HER2 + vs. HR + /HER2- (AOR = 1.35, p = 0.321), increased odds of being HR-/HER2 + vs. HR + /HER2- (AOR = 2.08, p = 0.023) and increased odds of being triple-negative (AOR = 2.67, p < 0.001) (Table 2). Tumour size was strongly associated with molecular subtypes. Compared to tumours > 5 cm, tumours ≤ 2 cm had equal odds of being HR + /HER2 + (AOR = 0.99, p = 0.978), significantly reduced odds of being HR-/HER2 + (AOR = 0.43, p = 0.027) and lower odds of being triple-negative (AOR = 0.47, p = 0.014) (Table 2). Lymph node status was not associated with molecular subtype. Histological type demonstrated a strong association with molecular subtype. Lobular histology compared to ductal histology had equal odds of being HR + /HER2 + subtype (AOR = 0.56, p = 0.411), equal odds of being HR-/HER2 + subtype (AOR = 0.16, p = 0.084) and reduced odds of being triple-negative (AOR = 0.17, p = 0.009) (Table 2). SBR grade was also associated with molecular subtype. Grade 1 tumours were predominantly HR + /HER2- (75%), while grade 3 tumours showed high rates of triple-negative disease (53%) (data not shown). Notably, HER2 + tumours (combining HR + /HER2 + and HR-/HER2 +) were most common in grade 2 (28%) and grade 3 (25%) tumours (data not shown). Compared to grade 1 tumours, grade 3 tumours had significantly increased odds of being HR + /HER2 + (AOR = 5.68, p < 0.001), HR-/HER2 + (AOR = 24.22, p < 0.001), or triple-negative (AOR = 13.92, p < 0.001) (Table 2). These findings align with previous studies showing strong correlations between tumour grade and molecular subtypes [34, 35].

**Table 2:** Multivariate Analysis of Clinicopathological Features Associated with Lymph Node Status and Molecular Subtypes

	HR + /HER2 + vs HR + /HER2-	HR-/HER2 + vs HR + /HER2-	Triple negative vs HR + /HER2-	Lymph node positive Vs. Negative
	AOR (95% CI)	AOR (95% CI)	AOR (95% CI)	AOR (95% CI)
Specimen				
Biopsy	1.00 [reference]	1.00 [reference]	1.00 [reference]	1.00 [reference]
Mastectomy	1.35 (0.75–2.45)	2.09 (1.10–3.95)*	2.67 (1.65–4.34)***	14.33 (8.67–23.69)***
Tumour Size				
Under 2 cm	0.99 (0.41–2.37)	0.43 (0.20–0.91)*	0.47 (0.26–0.86)*	0.24 (0.14–0.42)***
2–5 cm	1.06 (0.46–2.49)	0.45 (0.22–0.91)*	0.49 (0.28–0.87)*	0.40 (0.23–0.69)**
Over 5 cm	1.00 [reference]	1.00 [reference]	1.00 [reference]	1.00 [reference]
Histology				
Ductal	1.00 [reference]	1.00 [reference]	1.00 [reference]	1.00 [reference]
Lobular	0.56 (0.16–1.95)	0.16 (0.02–1.28)	0.17 (0.04–0.64)**	0.68 (0.29–1.55)
Other	1.7 (0.58–4.95)	1.01 (0.27–3.75)	1.84 (0.79–4.29)	1.01 (0.44–2.32)
SBR Grade				
1	1.00 [reference]	1.00 [reference]	1.00 [reference]	1.00 [reference]
2	2.71 (1.22–6.03)*	6.15 (1.84–20.58)**	1.63 (0.91–2.94)	1.90 (1.14–3.17) *
3	5.68 (2.26–14.28)***	24.22 (6.89–85.07)***	13.92 (7.31–26.5)***	1.49 (0.85–2.61)

^*^p < 0.05; **p < 0.01; ***p < *0.001; *OR: Odds ratio; CI: Confidence interval; HR: Hormone receptor; HER2: Human epidermal growth factor receptor 2; SBR: Scarff-Bloom-Richardson

Models adjusted for Specimen, Tumour size, Histology and SBR grade. Models adjusted for specimen, tumour size, histology, and SBR grade

This comprehensive table presents the results of multivariate logistic regression analyses, displaying adjusted odds ratios with 95% confidence intervals for the associations between clinicopathological features and both lymph node status and molecular subtypes. The table consolidates findings from multiple regression models into a single, easy-to-reference format. It quantifies the strength of associations between tumour characteristics and outcomes, revealing that mastectomy specimens had 14 times higher odds of lymph node positivity, tumours ≤ 2 cm had 75% lower odds of nodal involvement, and SBR grade 3 tumours had over 10 times higher odds of being triple-negative compared to grade 1 tumours. Asterisks indicate the level of statistical significance (*p < 0.05, **p < 0.01, ***p < 0.001). Both models were adjusted for specimen, tumour size, histology, and SBR grade

### Associations with lymph node status

Table 2 also presents the model estimated to explore factors associated with lymph node status. Tumour size showed a strong association with lymph node status, with tumours ≤ 2 cm having significantly lower odds of lymph node involvement compared to tumours > 5 cm (AOR = 0.24, p < 0.001) (Table 2), consistent with established understanding of size-nodal relationships [36]. SBR grade showed a significant association with lymph node status, with grade 2 tumours having approximately almost twice the odds of nodal involvement compared to grade 1 tumours (AOR = 1.90, p = 0.014 for both) (Table 2).

### Comparison with other populations

Table 3 presents a comparison of triple-negative breast cancer rates across different populations. Our observed TNBC rate of 27% in Jamaican women is identical to rates reported in Nigerian and Senegalese populations (27%) [19] and similar to rates in Barbadian women (25%) [22], but substantially lower than the high rates observed in Ghanaian women (53–58%) [20, 37]. Compared to populations in the United States, our TNBC rate exceeds those reported for non-Hispanic white women (10.8%) and the overall U.S. population (12.8%) [6, 38], but is comparable to rates among non-Hispanic Black women (24.6%) [38]. These variations highlight the unique molecular profile of breast cancer across different populations of African ancestry and underscore the importance of population-specific approaches to breast cancer management.

**Table 3:** Comparison of Triple-Negative Breast Cancer Rates Across Different Populations

Author	Year	Location	Sample Size	TNBC (%)
Giaquinto et al. [6]	2024	United States	118,947	12.8
Bauer et al. [38]	2007	California, USA	51,074	Overall: 12.5 Non-Hispanic Black: 24.6 Non-Hispanic White: 10.8
Jiagge et al. [20]	2016	Michigan, USA	593	African American: 29.8 White American: 15.5
Huo et al. [19]	2009	Nigeria and Senegal	507	27.0
Deloumeaux et al. [21]	2017	Guadeloupe	1275	14.0
Hercules et al. [22]	2020	Barbados	842	25.0
Der et al. [37]	2015	Ghana	223	58.0

TNBC: Triple-negative breast cancer

This comparative table positions the study's findings within the global context by presenting the prevalence of triple-negative breast cancer (TNBC) across diverse populations. It includes data from nine studies conducted between 2007 and 2024 in various geographical regions, including the United States, Caribbean islands, and African countries. The table highlights significant variations in TNBC rates, ranging from 10.8% in non-Hispanic White Americans to 58% in Ghanaian women. The current study's TNBC rate of 27% in Jamaican women aligns more closely with findings from other Black populations, including those from Nigeria and Senegal (27%) and Barbados (25%), while differing substantially from the higher rates observed in Ghana and the lower rates reported in Guadeloupe and among non-Hispanic White Americans

## Discussion

This study provides the most comprehensive analysis to date of molecular subtypes of breast cancer in a Jamaican cohort, offering critical insights into the distinct patterns of disease in this predominantly Black Caribbean population. Our findings have important implications for breast cancer screening, diagnosis, and treatment approaches in Jamaica and similar Caribbean settings.

The distribution of molecular subtypes in our Jamaican cohort reveals several noteworthy findings. While HR + /HER2- tumours constitute the largest group (49%), we observed a remarkably high prevalence of both triple-negative breast cancer (TNBC) (27%) and HER2 + disease (25%). This molecular profile differs substantially from patterns in predominantly White populations but shares similarities with other Black populations globally. The 27% TNBC rate in our cohort is comparable to frequencies observed in Nigerian and Senegalese populations (27%) [39] and Barbadian women (25%) [22], but considerably higher than rates reported in White Americans (10.8%) [40] and the general U.S. population (12.8%) [6]. Our TNBC prevalence is lower than the extraordinarily high rates documented in Ghanaian women (53–58%) [20, 37], suggesting that while shared African ancestry influences TNBC risk, additional factors—potentially including admixture patterns, environmental exposures, or lifestyle factors—may moderate this risk in Caribbean populations.

Increased TNBC incidence has been noted in populations with high frequency of expression of *BRCA*, *Kaiso*, and *PALB2* genes, a characteristic common to both Caribbean and African breast cancer groups. The use of skin lighteners and hair relaxers is a lifestyle practice limited to pigmented populations and is common among women of African descent. The Jamican Health & Lifestyle Survey 2016–2017 implies that ~ 9% of Jamaican women report using skin lighteners. These agents often contain toxic heavy metal compounds and phthalates, both of which associated with carcinogenesis. Particularly striking is our finding of a 25% prevalence of HER2 + positive tumours (combining HR + /HER2 + and HR-/HER2 + subtypes), which exceeds the typically reported 15–20% in most populations [30]. The 25% prevalence of HER2-positive disease has significant clinical and healthcare policy implications for Jamaica. HER2-positive tumors are associated with rapid growth, increased tumor spread, and higher recurrence rates compared to hormone receptor-positive or triple-negative cancers when left untreated. While anti-HER2 therapy (trastuzumab) became available through the National Health Fund as first-line treatment from 2015, access remains limited to first-line therapy due to cost constraints. This high prevalence of HER2-positive disease underscores the urgent need for improved access to targeted therapies and may require healthcare policy adjustments to ensure adequate resource allocation for anti-HER2 treatments in the Jamaican healthcare system. The higher proportion of HER2 + tumours in our population suggests a critical need for ensuring access to HER2 testing and targeted therapies in Jamaica to improve survival outcomes, as HER2 targeted therapies have dramatically improved outcomes for patients with HER2 + disease [41].

Our analysis revealed strong associations between molecular subtypes and clinicopathological features. HR + /HER2- tumours demonstrated favorable characteristics, including smaller tumour size, lower grade, and reduced odds of lymph node involvement. Conversely, TNBC and HER2 + tumours exhibited more aggressive features, including larger tumour size, higher grade, and increased nodal involvement. The association between SBR grade and molecular subtype was particularly strong. Grade 3 tumours had more than ten-fold the odds of being TNBC compared to grade 1 tumours (AOR = 13.92), consistent with the known aggressive biology of TNBC [34]. Similarly, HER2 + tumours were significantly more likely to be higher grade (grades 2 and 3), aligning with the established understanding of HER2 + disease as biologically aggressive [35].

Interestingly, we observed no significant association between molecular subtypes and age at diagnosis, contrasting with numerous studies that report younger age for triple-negative breast cancer. Triple-negative breast cancer accounts for about 10–15% of all breast cancers and tends to be more common in women younger than age 40, who are Black, or who have a BRCA1 mutation. Studies have shown that TNBC constitutes a clinically challenging type of breast cancer that occurs more frequently in younger women (< 50 years) and African American women [42, 43]. Population-based studies from the United States have consistently demonstrated that younger women have significantly higher odds of triple-negative diagnosis, with women in the youngest age groups showing markedly elevated risks [40, 42]. This lack of age-related differences in our Jamaican population may reflect unique epidemiological patterns related to genetic ancestry, environmental factors, or the underlying biology of breast cancer in Caribbean populations. Previous studies have shown that women of certain races/ethnicities, including those of African descent, have 1.4- to 3.1-fold elevated risks of presenting with estrogen receptor-negative/progesterone receptor-negative breast cancer compared to non-Hispanic whites, suggesting that biological and lifestyle factors contribute to population-specific tumor characteristics [42, 44]. The absence of the typical age-ER association seen in other populations warrants further investigation and may suggest different tumor development pathways in women of African Caribbean descent.

Jamaica faces a particularly concerning pattern of breast cancer mortality, with rates of 28.3 per 100,000 reported in 2014 [5], significantly higher than those observed in developed countries. Our findings suggest that the molecular profile of breast cancer in Jamaican women may contribute to these elevated mortality rates through two mechanisms: the high prevalence of aggressive molecular subtypes (TNBC and HER2 +) and potential disparities in access to appropriate targeted therapies. The 27% prevalence of TNBC is especially concerning given the limited treatment options and poorer outcomes associated with this subtype [15]. TNBC is not amenable to endocrine therapy or HER2 targeted treatment, leaving chemotherapy as the primary systemic approach. Additionally, the 25% prevalence of HER2 + disease highlights the importance of ensuring access to anti-HER2 therapies in Jamaica. Without these targeted treatments, patients with HER2 + disease face significantly worse outcomes [45]. Our findings support the implementation of universal hormone receptor and HER2 testing for all breast cancer patients in Jamaica, as recommended by international guidelines [33]. Additionally, they underscore the need for focused efforts to increase access to targeted therapies, particularly anti-HER2 agents, which have demonstrated remarkable efficacy in improving outcomes for patients with HER2 + disease [33, 46]. The observed associations between molecular subtype and clinicopathological features also have implications for screening and early detection strategies. The predominance of HR + /HER2- tumours among smaller, lower-grade lesions underscores the benefit of mammographic screening for detecting these cancers at earlier stages. However, the significant proportion of aggressive subtypes suggests that additional approaches may be needed to effectively detect rapidly growing tumours, which can develop between screening examinations [47].

Our study has certain limitations. As a retrospective analysis from a single institution, generalizability should be considered in context, though UHWI serves as a major referral center for 10 of Jamaica's 14 parishes. Missing data for tumor size (33%) and SBR grade (30%) exists, but sensitivity analyses revealed no significant differences between complete and incomplete cases. Bivariate tests of association revealed no significant difference in the distribution of molecular subtype between missing values and non-missing values. While we used clinically standard IHC surrogates rather than gene expression profiling for molecular classification, this approach aligns with routine clinical practice worldwide [48](23). These limitations are offset by notable strengths. Our study represents the largest analysis of breast cancer molecular subtypes in Jamaica to date (n = 1312) over a five-year period. The standardized IHC methods following international guidelines enhance reliability, and our comprehensive analysis of associations between molecular subtypes and clinicopathological features provides valuable insights into breast cancer patterns in the Jamaican population that can inform clinical practice and future research.

## Conclusion

Our study demonstrates that breast cancer in Jamaican women exhibits a distinct molecular profile characterized by a high prevalence of aggressive subtypes, particularly triple-negative (27%) and HER2 positive (25%) disease. These findings have significant implications for breast cancer management in Jamaica, highlighting the need for universal receptor testing, access to targeted therapies, and tailored screening strategies. Our results contribute to the understanding of breast cancer heterogeneity across populations of African ancestry and underscore the importance of population-specific approaches to breast cancer prevention, detection, and treatment in the Caribbean region.

## Supplementary Information

Below is the link to the electronic supplementary material.

Supplementary Material 1 Supplemental Excel file was purposefully empty to adhere to the data protection guidelines as per the UWI institutional ethics letter or approval.

## Data Availability

The datasets used and analyzed during the current study are available from the corresponding author on reasonable request, subject to approval by the Faculty of Medical Sciences Ethics Committee at the University of the West Indies.
